# Comprehensive modeling of cell culture profile using Raman spectroscopy and machine learning

**DOI:** 10.1038/s41598-023-49257-0

**Published:** 2023-12-09

**Authors:** Hiroki Tanemura, Ryunosuke Kitamura, Yasuko Yamada, Masato Hoshino, Hirofumi Kakihara, Koichi Nonaka

**Affiliations:** 1 Biologics Technology Research Laboratories I, Biologics Division, Daiichi Sankyo Co., Ltd., 2716-1, Aza Kurakake, Oaza Akaiwa, Chiyoda-Machi, Oura-Gun, Gunma, 370-0503 Japan; 2 Analytical & Quality Evaluation Research Laboratories, Pharmaceutical Technology Division, Daiichi Sankyo Co., Ltd., 1-12-1, Shinomiya, Hiratsuka, Kanagawa 254-0014 Japan; 3 Biologics Division, Daiichi Sankyo Co., Ltd., 2716-1, Aza Kurakake, Oaza Akaiwa, Chiyoda-Machi, Oura-Gun, Gunma, 370-0503 Japan

**Keywords:** Raman spectroscopy, Antibody therapy, Machine learning

## Abstract

Chinese hamster ovary (CHO) cells are widely utilized in the production of antibody drugs. To ensure the production of large quantities of antibodies that meet the required specifications, it is crucial to monitor and control the levels of metabolites comprehensively during CHO cell culture. In recent years, continuous analysis methods employing on-line/in-line techniques using Raman spectroscopy have attracted attention. While these analytical methods can nondestructively monitor culture data, constructing a highly accurate measurement model for numerous components is time-consuming, making it challenging to implement in the rapid research and development of pharmaceutical manufacturing processes. In this study, we developed a comprehensive, simple, and automated method for constructing a Raman model of various components measured by LC–MS and other techniques using machine learning with Python. Preprocessing and spectral-range optimization of data for model construction (partial least square (PLS) regression) were automated and accelerated using Bayes optimization. Subsequently, models were constructed for each component using various model construction techniques, including linear regression, ridge regression, XGBoost, and neural network. This enabled the model accuracy to be improved compared with PLS regression. This automated approach allows continuous monitoring of various parameters for over 100 components, facilitating process optimization and process monitoring of CHO cells.

## Introduction

In recent years, the development of monoclonal antibodies (mAbs) using genetic recombination techniques has garnered increasing attention given the potential of these agents regarding their high specificity and efficacy. Since the approval of muromonab-CD3 in 1986, antibody-based drugs have been predominantly developed for cancer and autoimmune diseases, with over 120 drugs approved by 2021^1^. Chinese hamster ovary cells (CHO cells) are the primary choice for manufacturing antibody drugs, and efforts have been made to develop a stable antibody production process^2^. The productivity of antibodies in CHO cells significantly impacts the cost of production and stability of supply. Ensuring production of the required quantity in a single production run is desirable, especially considering the limited production facilities available. With the demand for antibody drugs growing each year, there is a strong societal need to improve their productivity.

In biopharmaceutical production, there are two main methods for culturing CHO cells: fed-batch culture and perfusion culture. When employing either of these methods, it is crucial to appropriately monitor and control key factors to achieve the high production of high-quality antibodies^3^. Medium components such as glucose and amino acids, along with various metabolites, play a significant role in the productivity and quality of antibodies. Analyzing and managing the concentrations of these factors is known to improve antibody productivity^4^. Oxidative and endoplasmic reticulum stress during cell culture may also impact antibody productivity^5^. Previously, we identified the Hspa5 promoter, whose expression of antibodies is suggested to be directly affected by endoplasmic reticulum stress^6^. It is important to note that the factors related to stress are not limited to a single factor, but rather several factors. Therefore, monitoring stress markers comprehensively is especially important for this type of promoter.

At present, to monitor cell culture profile during production culture, small samples of medium components and metabolites are taken at certain culture points and quantified using a bioanalyzer or LC–MS. However, this sampling process poses challenges, including potential effects on the culture volume and the risk of microbial contamination. Moreover, the limited number of sampling points makes it difficult to obtain data at high frequencies. Consequently, various process analytical technology (PAT) methods have been developed for continuous analysis. For example, Raman spectrometers and near-infrared spectroscopy can provide information on components in the culture solution, while capacitance-based measurements enable cellular concentration analysis^7^. In recent years, the utilization of Raman spectrometers has been explored not only in cultivation processes but also in purification processes to maintain process consistency^8^.

In this study, we focused on the potential of Raman spectrometry as a method for continuous analysis. Raman spectroscopy equipment generates Raman scattering light by irradiating laser light onto a sample, which carries information on the inherent oscillation frequency of molecules^9–11^. By detecting this light, the concentration of specific molecules can be measured. In previous studies, Raman measurement systems were developed for primary medium components and metabolite concentrations, including glucose, lactate, and amino acids^12,13^. Additionally, feedback control systems have been established to achieve optimal concentrations^14–17^. Moreover, it has been reported that the constructed model is scalable across different culture scales^18^. Reports of measuring cell growth, pH, and antibody quality, as well as applications in perfusion culture, have also been published^19–24^. Furthermore, equipment for acquiring Raman spectra using small-scale reactors has been commercialized and is effective for constructing models that require a large number of data points^25,26^.

Partial least square (PLS) regression is commonly employed as a model-building method, which selects principal components to capture a linear relationship between predictor and response variables. It allows the construction of more accurate models by reducing explanatory variables through dimensionality reduction. Spectral range selection methods were previously reported such as manual selection based on prior knowledge, stepwise selection, and genetic algorithms^27–29^. However, optimizing the model construction conditions, such as spectrum range and pretreatment of spectrum data, to enhance accuracy involving a time-consuming process of trial and error. This makes it challenging to construct highly accurate models for numerous measurement items within the rapid research and development context of pharmaceutical manufacturing processes.

Recently, machine learning has emerged as a method for improving model construction accuracy, and some studies have reported its application in analyzing Raman spectral data^30–34^. By incorporating machine-learning techniques into Raman measurement model construction for culture profiles, it is possible to efficiently construct highly accurate models, even when PLS regression fails to produce high-performance models.

In this study, we focused on measuring broad range of categories of parameters and developed a comprehensive, simple, and automated method for constructing an exhaustive Raman model using machine learning in Python. This method enabled convenient and high-throughput data acquisition for Raman model construction by utilizing a small automated culture vessel. As we aimed to comprehensively construct models for a large number of components, we employed Bayesian optimization for optimizing the preprocessing, spectral range and hyperparameter. Bayesian optimization is a method that estimates the global optimum of a function by learning unknown functions from data using Gaussian process regression^35^, while minimizing the number of trial iterations and make it easy to construct suitable model for various components. Subsequently, models were constructed for each component using various model construction techniques, such as linear regression, ridge regression, XGBoost, and neural network, and their accuracies were compared.

## Results

### Automated and comprehensive construction of optimal Raman models by PLS regressions with Bayes optimization

To construct a Raman measurement model for data from various cell cultures, we initially cultured CHO cells using multiple small culture vessels (Ambr250), sampled them over time, and measured the levels of metabolites and medium components. Raman spectra were acquired using the Spectroscopy module at the same time as sampling. To obtain results from diverse cultures, three strains were used as cell clones, and fed-batch cultures were performed in duplicate. Several analyses were performed, including metabolite analysis using a bioanalyzer, antibody concentration analysis by HPLC, cell concentration and cell viability analyses by ViCEll, and metabolite component analysis by LC–MS, to comprehensively acquire data from various cultures (Supplemental Fig. 1).

**Figure 1:**
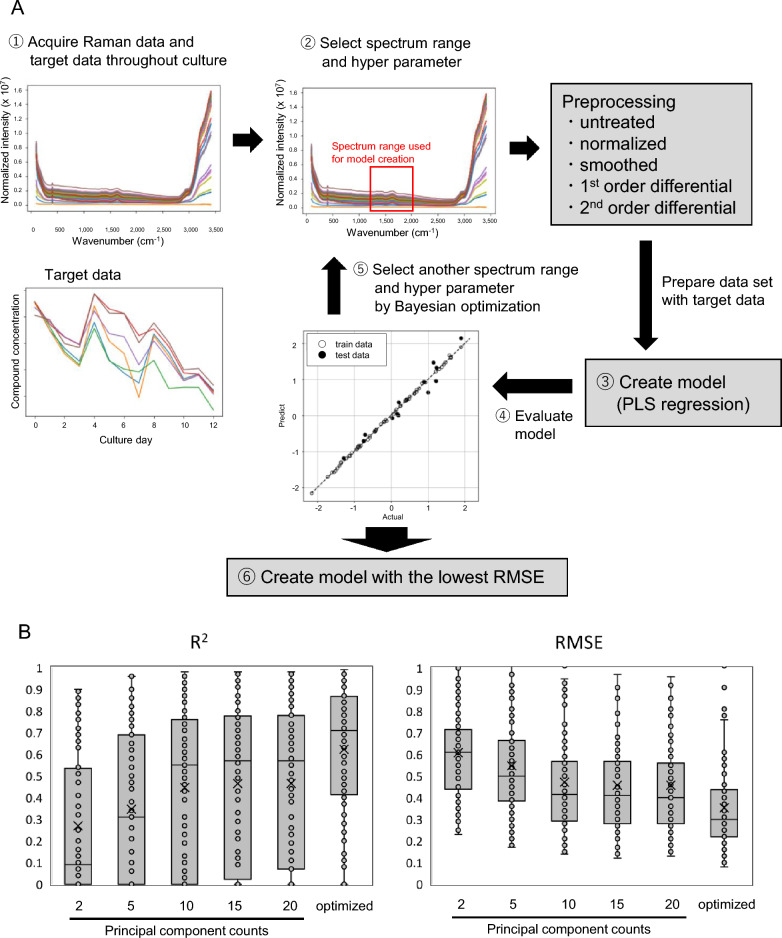
Model-optimized transition scheme and optimization result. (**A**) Model-building conditional optimization scheme for PLS regressions. (1) Specifying the spectral regions used for model construction. (2) Generating datasets for the specified spectral regions and preprocessing them in the order of untreated, normalized, smoothed (moving average of 10 points), first-order differential, and second-order differential. (3: Creating a dataset that includes the preprocessed spectral data, along with the data of the component density for model construction (after normalization). The dataset was divided into learning and test data, and PLS regression was performed. Principal component counts for PLS regressions ranged from 0 to 15, with five of the six reactor datasets used as learning data and one as verification data. (4) Creating a model by PLS regression using Python machine learning library (scikit-learn). (5) Evaluating the model performance using the test data and calculating R^2^ and root mean squared error (RMSE) as indicators. (6) Changing the principal component numbers of the spectral domain and PLS regression and performing PLS regression with the Bayes optimization specifying the parameters to be examined next. (7) Repeating the above steps and calculating the spectral range, pretreatment conditions, and principal component numbers of PLS regressions where RMSE was minimal when the test data were applied to the model. (**B**) To verify the effect of model optimization, the model performance was compared with the case where the model was constructed without optimization. The full spectral domain without preprocessing was used for the spectra, and n_components was set to 2, 5, 10, 15 and 20. R^2^ and RMSE of the constructed models were plotted, and the means and error ranges were compared using a box-and-whisker plot. In this plot, an "x" (cross) represents the mean, the box represents the interquartile range, a line inside the box represents the second quartile, and the whiskers represent the minimum (1.5 times the interquartile range below the first quartile) and maximum (1.5 times the interquartile range above the third quartile) values of the data.

Subsequently, a Raman measurement model for data from various cultures was constructed using PLS regression from the obtained culture data and Raman spectral data. The spectral domain used for model construction, hyperparameter (n_components) of PLS regression analysis, and optimization of data preprocessing methods were performed following the scheme shown in Fig. 1A. The process involved the following steps. (1) Specifying the spectral regions used for model construction. (2) Generating datasets for the specified spectral regions and preprocessing them in the order of untreated, normalized, smoothed (moving average of 10 points), first-order differential, and second-order differential. (3) Creating a dataset that includes the preprocessed spectral data, along with the data of the component density for model construction (after normalization). The dataset was divided into learning and test data, and PLS regression was performed. Principal component counts for PLS regressions ranged from 0 to 15, with five of the six reactor datasets used as learning data and one as verification data. (4) Creating a model by PLS regression using Python machine-learning library (scikit-learn). (5) Evaluating the model performance using the test data and calculating R^2^ and root mean squared error (RMSE) as indicators. (6) Changing the principal component numbers of the spectral domain and PLS regression and performing PLS regression with the Bayes optimization specifying the parameters to be examined next. (7) Repeating the above steps and calculating the spectral range, pretreatment conditions, and principal component numbers of PLS regressions where RMSE was minimal when the test data were applied to the model.

Raman models for data from various cultures were constructed using the above methods, the results of which are presented in Table 1. When examining the model accuracy by compound group, a model with an R^2^ greater than 0.5 was constructed for almost all amino acids. Interestingly, models with high measurement accuracy were also constructed for theoretically undetectable components, such as metal ions, oxygen, and carbon dioxide. However, for vitamins, approximately half of the components that could be modeled and constructed had an R^2^ below 0.5. The modeling accuracy for metabolites (carbohydrate metabolism, amino acid metabolism, nucleic acid metabolism) with an R^2^ above 0.5 was approximately half. These results demonstrate the successful construction of Raman measurement models with fixed precision for each compound group. Comparing the model accuracy between different preprocessing methods revealed variations in model accuracy (Table 2).

**Table 1:** Model outcomes (PLS regression) for individual components.

Target	Category	Spectrum_min (cm^−1^)	Spectrum_max (cm^−1^)	n_components	Processing	R^2^	RMSE
Pantothenic acid	Vitamin	2848	3425	9	Raw	0.98	0.16
Fumaric acid	Vitamin	393	3425	15	Raw	0.89	0.27
Nicotinic acid	Vitamin	698	2045	9	Smoothed	0.86	0.34
4-Pyridoxic acid	Vitamin	614	3425	3	1st derivative	0.85	0.35
Cyanocobalamin	Vitamin	2831	3425	1	Raw	0.83	0.40
Orotic acid	Vitamin	2221	3425	11	Raw	0.81	0.45
Hexose (glucose)	Sugar	637	3255	14	Raw	0.93	0.23
Titer	Protein	514	3407	11	Smoothed	0.96	0.19
Hspa5 (mg/mL)	Protein	805	1205	12	Smoothed	0.84	0.45
Glycyl-glutamine	Peptide	881	2964	7	Smoothed	0.93	0.21
Viability	Others	945	3425	6	Raw	0.95	0.20
Osmolarity	Others	776	3009	9	Smoothed	0.95	0.24
Viable cell density	Others	183	3036	15	Raw	0.82	0.32
pH	Others	908	3322	12	Raw	0.80	0.33
Uridine monophosphate	Nucleic acid	839	1470	7	Raw	0.98	0.13
Guanine	Nucleic acid	1709	2715	6	2nd derivative	0.94	0.16
Deoxycytidine monophosphate	Nucleic acid	100	2965	13	Raw	0.88	0.27
Deoxyadenosine monophosphate	Nucleic acid	2444	3425	1	1st derivative	0.86	0.34
Adenosine monophosphate	Nucleic acid	642	3425	15	1st derivative	0.84	0.34
Inosine	Nucleic acid	1417	3214	14	Smoothed	0.83	0.25
Malic acid	Metabolite	517	2515	15	Smoothed	0.97	0.18
Succinic acid	Metabolite	8D7	18D2	8	Raw	0.95	0.23
Lactic acid	Metabolite	159	1270	8	Raw	0.88	0.24
Ornithine	Metabolite	118	918	15	Smoothed	0.84	0.34
3-Aminopropanoic acid	Metabolite	219	2870	6	Smoothed	0.81	0.25
K^+ ^	Ion	840	1959	10	Smoothed	0.95	0.20
NH^4+ ^	Ion	682	3425	12	Raw	0.93	0.26
Na^+ ^	Ion	687	3425	11	Smoothed	0.86	0.27
Ca^2+ ^	Ion	1205	3425	9	Raw	0.81	0.41
4-Hydroxyproline	Amino acid metabolite	942	2241	15	Smoothed	0.99	0.11
3-Methylhistidine	Amino acid metabolite	642	2349	6	Raw	0.96	0.15
Argininosuccinic acid	Amino acid metabolite	1170	3425	15	Raw	0.94	0.20
3-Methyl-2-oxovaleric acid	Amino acid metabolite	192	1802	12	Smoothed	0.92	0.30
2-Aminoadipic acid	Amino acid metabolite	2085	3425	2	Raw	0.89	0.35
Saccharopine	Amino acid metabolite	1404	3425	10	Smoothed	0.88	0.33
Kynurenic acid	Amino acid metabolite	232	956	11	Raw	0.87	0.35
Citrulline	Amino acid metabolite	1100	3425	7	Raw	0.85	0.37
5-Oxoproline	Amino acid metabolite	1955	3425	7	Raw	0.83	0.50
Kynurenine	Amino acid metabolite	642	1085	9	Smoothed	0.80	0.39
Methionine	Amino acid	708	3354	12	Raw	0.97	0.19
Tyrosine	Amino acid	818	1238	5	Raw	0.96	0.24
Threonine	Amino acid	828	3425	7	1st derivative	0.94	0.22
Aspartic acid	Amino acid	1887	3289	6	Raw	0.93	0.27
Phenylalanine	Amino acid	839	1438	15	Raw	0.93	0.27
Asparagine	Amino acid	658	1433	15	Smoothed	0.91	0.27
Glycine	Amino acid	2735	3425	12	Raw	0.91	0.25
Tryptophan	Amino acid	100	2905	12	Raw	0.87	0.33
Histidine	Amino acid	100	3425	15	Raw	0.86	0.37
Glutamine	Amino acid	727	1345	15	Raw	0.83	0.42
Serine	Amino acid	710	1220	15	Raw	0.82	0.34

Comprehensive models were constructed using PLS regression. The table shows data with an R^2^ of 0.8 or higher. The table includes compound groups, lower and upper limits of the spectral range used for model construction, principal component numbers used for PLS regression, spectral preprocessing techniques, and model performance (R^2^, RMSE).

**Table 2:** Comparing modeling accuracy with different preprocessing methods.

Evaluation	Raw	Standardized	Smoothed	1st derivative	2nd derivative
R^2^	0.93	0.93	0.9	0.9	− 4.31
RMSE	0.23	0.23	0.28	0.29	2.06

Model performance (R^2^, RMSE) when constructing glucose densitometry models with PLS regressions using various preprocessing techniques (untreated, standardized, smoothed, first-order differential, and second-order differential) is shown.

Furthermore, to verify the effect of optimizing the model construction conditions, the model performance was compared to the case where the conditions were not optimized. The spectra used the full spectral domain without preprocessing, and n_components was set to 2, 5, 10, 15 and 20. The constructed models’ R^2^ and RMSE were plotted, and the mean and error ranges were compared using box-and-whisker plots. The results demonstrated that the average model accuracy increased with the higher n_components value and optimizing the model construction conditions improved average R^2^ to 0.62 and decreased average RMSE to 0.35, indicating the significant enhancement of model performance (Fig. 1B).

**Figure 2:**
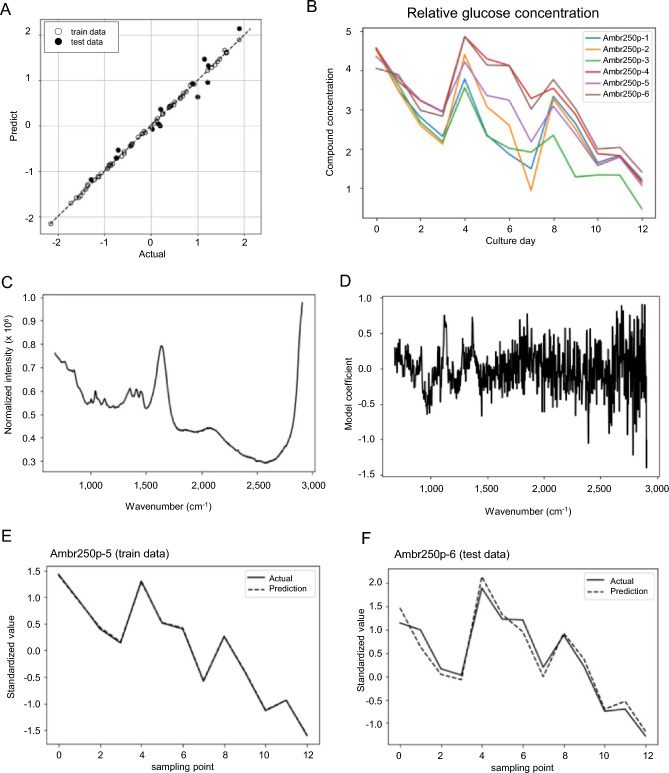
Model outcomes under optimized conditions. Results of constructing a Raman model under optimal conditions for measuring glucose. (**A**) The observed values of glucose concentration and the predicted values from the constructed Raman measurement model were plotted to confirm the correlation. Open circles represent training data and closed circles represent test data. (**B**) Time course of glucose concentration in each culture vessel. Ambr250p-1 to 6 indicate the culture vessel names, respectively. (**C**) One representative dataset of the spectrum used for model construction. (**D**) Model coefficients for each wavenumber in the Raman spectrum. (**E**) Observed value (solid line) and predicted value (dotted line) of glucose levels at Ambr250p-5 used for the training data. (**F**) Observed value (solid line) and predicted value (dotted line) of glucose levels at Ambr250p-6 used for the test data.

To visualize the accuracy of measurement of each component, glucose concentration was used as an example. The plots of predicted and actual measurements, the time course of glucose concentration in each culture vessel (actual measurements), the used spectral regions (pretreated), and the modeling factors are shown in Fig. 2A–D. Comparison between the actual time course and prediction results revealed similar transitions in the learning and test data, indicating the construction of a highly accurate prediction model, with R^2^ of 0.93 and RMSE of 0.23 (Fig. 2E,F).

### Comparison of machine-learning methods

**Figure 3:**
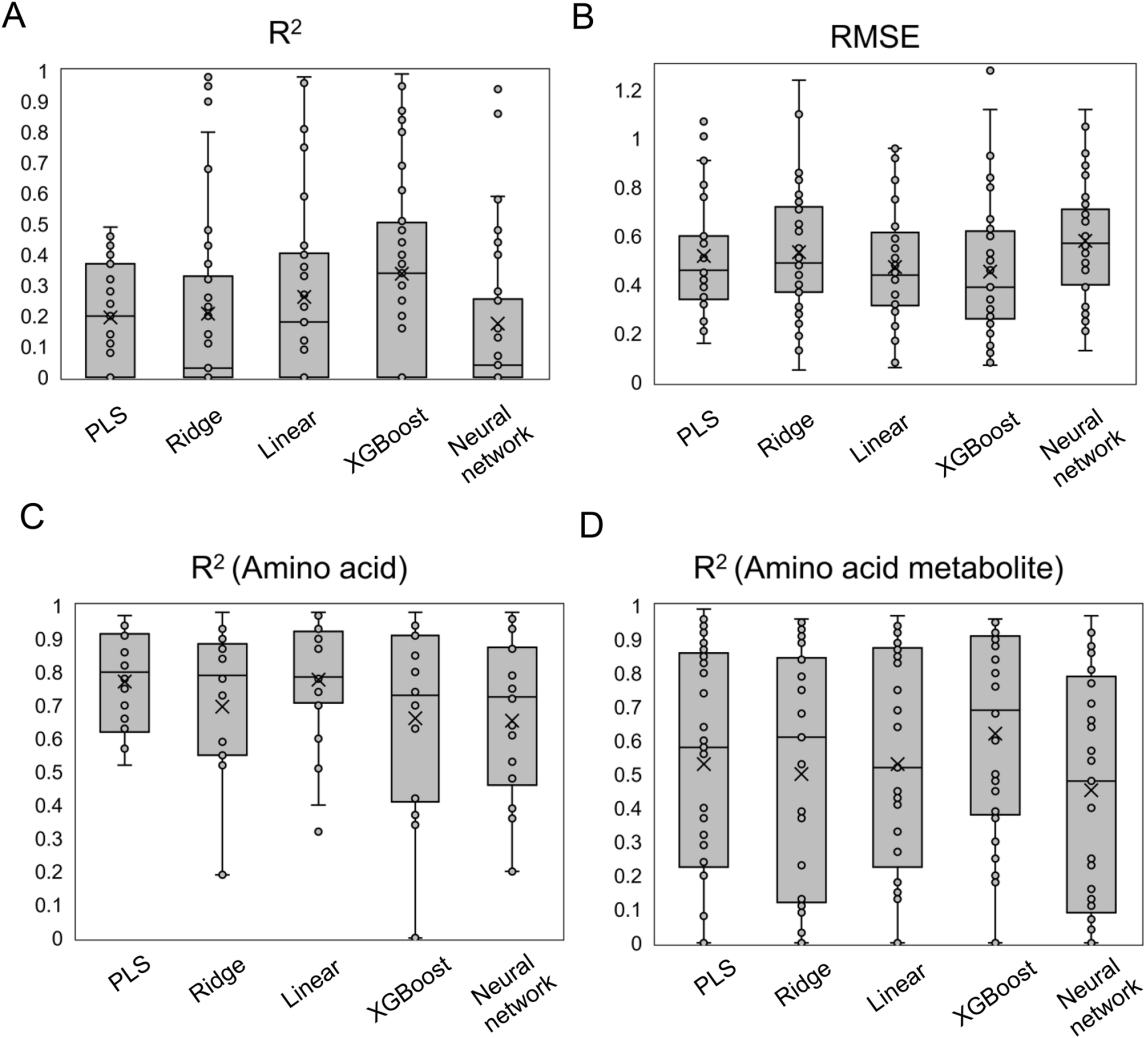
Comparing the performance of Raman measurement models with different machine-learning techniques. Raman measurement models were constructed for each parameter using various machine-learning techniques (PLS regression, ridge regression, linear regression, XGBoost, and neural network), and their performance was evaluated. For the target component with an R^2^ value less than 0.5 in the PLS model, the R^2^ value (**A**) and RMSE (**B**) were plotted, and the means and error ranges were shown using box-and-whisker plots. The R^2^ values of the models for amino acids (**C**) and amino acid metabolites (**D**) were also plotted, and the means and error ranges were shown using box-and-whisker plots. In this plot, an "x" (cross) represents the mean, the box represents the interquartile range, a line inside the box represents the second quartile, and the whiskers represent the minimum (1.5 times the interquartile range below the first quartile) and maximum (1.5 times the interquartile range above the third quartile) values of the data.

To assess the improvement of accuracy of the model, we examined machine-learning methods other than PLS regression using a similar approach. Linear regression and ridge regression, commonly used as regression analysis methods, were considered as model construction methods. For ridge regression, optimization of the hyperparameter α was also performed. XGBoost and neural network were validated as machine-learning techniques. Default hyperparameters for scikit-learn in Python were used. Raman-measuring models for each component were constructed using these machine-learning techniques. R^2^, RMSE values of the constructed models were plotted, and the means and error ranges were compared using box-and-whisker plots (Fig. 3).

Regarding the target component with an R^2^ value less than 0.5 in the PLS model, it was demonstrated that the R^2^ improved and RMSE decreased for several components when using another modeling method (Fig. 3A,B). When considering all components, regardless of the R^2^ value in the PLS model, the average R^2^ of certain categories, such as amino acid metabolites and vitamins, improved with XGBoost, while others, like amino acids, did not show a clear improvement. This suggests that the effect of changing the modeling method depends on the category of the target compound (Fig. 3C,D, Supplemental Fig. 2).

### Improved modeling of low-concentration protein by machine learning

**Figure 4:**
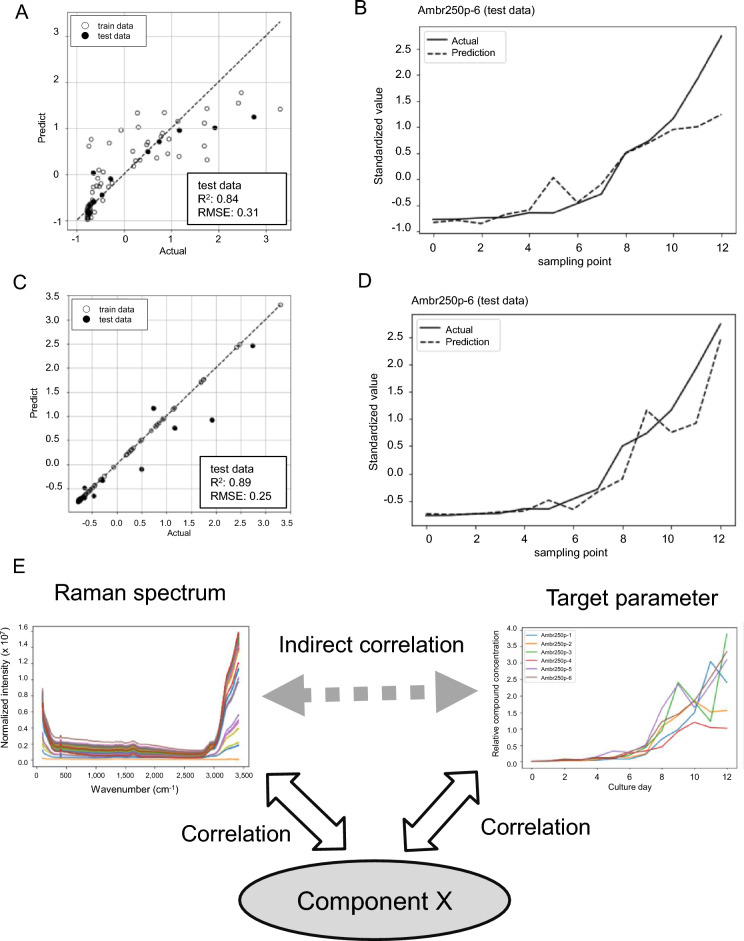
Improved modeling of low-concentration protein by machine learning and hypothetical indirect prediction model. Results of constructing a Raman model under optimal conditions for measuring BiP, an ER stress marker. (**A**) The actual measured BiP levels and the values predicted from the Raman measurement model constructed by PLS regression were plotted to confirm the correlation. Open circles represent training data and closed circles represent test data. (**B**) Actual measured glucose levels at Ambr250p-6 used for the test data (solid line) versus predicted PLS regressions (dotted line). (**C**) The actual measured BiP level and the value predicted from the Raman measurement model constructed by XGBoost were plotted to confirm the correlation. Open circles represent training data and closed circles represent test data. (**D**) Actual measured glucose levels (solid line) versus predicted XGBoost (dotted line) at Ambr250p-6 used for the test data. (**E**) Hypothesis explaining how each parameter can be predicted from Raman spectra. It is considered that the measurement object is correlated with a certain compound, and the compound concentration is indirectly measured through the Raman spectrum.

Based on the previous results, which demonstrated the successful construction of measurement models for medium components, metabolites, cell proliferation, and product concentration, we aimed to determine whether the measurement scope could be extended further. As an example, we examined whether a measurement model for BiP protein, a marker of endoplasmic reticulum stress, could be constructed^36^. Monitoring endoplasmic reticulum stress could be useful as it may inhibit protein production and worsen antibody quality in cell culture. The model construction conditions for PLS regression analysis were optimized following the same method as before, resulting in the construction of a Raman measurement model with an R^2^ of 0.84 and RMSE of 0.31 (Fig. 4A). There was noticeable variation between the plots of actual and predicted values for the training data. Although there were greater deviations at the end of culture when comparing the actual (ELISA) and predicted BiP levels over time, the general consistency between the predicted and observed levels for up to 10 days of culture suggested the ability to predict the timing of increase of BiP levels (Fig. 4B). The modeling was then performed using linear regression, ridge regression, XGBoost, and neural network in a similar manner. Upon model construction with XGBoost, the deviation between actual and predicted values improved, and the assessment using the test data resulted in an R^2^ of 0.89 and RMSE of 0.25 (Fig. 4C). The observed and predicted BiP levels over time showed overall consistency throughout the culture (Fig. 4D).

## Discussion

In this study, we developed a Python program that automates the optimization of principal component numbers in the spectral domain and PLS regression for a wide range of target compounds. We used PLS regression as an example for model construction. These conditions indicate whether model accuracy increases or decreases, and Bayesian optimization proves to be a powerful technique for optimization with low computational complexity. Particularly when optimizing the model for multiple targets, automation and computational speed are crucial, making it suitable for constructing models for various components. Furthermore, we applied the same method to linear regression, ridge regression, XGBoost, and neural network, demonstrating the versatility of the optimization of PLS regression modeling. This showcases the usefulness of Python programming in selecting the optimal model-building conditions.

In this study, we utilized the Raman sampling module of a small-scale culture vessel Ambr 250 to acquire Raman spectral data. Constructing a Raman model requires a large number of data points. Conventionally, a Raman spectrometer is inserted into a glass vessel to acquire data, but this method is time-consuming, as data on only one culture can be acquired per sensor. In contrast, our approach allows the simultaneous acquisition of Raman spectral data from multiple cultures, enabling the construction of Raman models using data from a single culture. This method proves to be a simple and efficient approach for constructing Raman models, aligning the Raman spectrum with the drug development timeline, and serving as a valuable monitoring method in process development and GMP production. In this study, we constructed models using Raman spectra measured in a microfluidic channel. It was reported that comparable models can be created using this measurement technique and by directly measuring with sensors within the bioreactor^25^. However, it is important to consider the potential for heterogeneity in the vessel, such as dissolved oxygen, when scaling up from fluid analysis^37^. Even when using Raman sensors to measure compound concentrations, it is necessary to consider this heterogeneity. Also, for model validation, we used five reactors for model construction and the remaining one reactor as a test dataset to evaluate the predictive accuracy. This was done to clearly observe the time-course changes in the predicted data of the test dataset, as shown in Fig. 2F. However, when actually constructing models using this method, even higher accuracy models may be built by using cross-validation, where test data is randomly sampled from the data of all reactors.

PLS regression is commonly used for constructing Raman models. Datasets of Raman spectra contain numerous explanatory variables, making them suitable for regression methods that involve dimensionality reduction, such as PLS regression. PLS regression has the advantages of high speed and comprehensive model construction for each measured object. In this study, we also examined linear regression, ridge regression, XGBoost, and neural network. Some machine-learning techniques exhibited modeling performance surpassing that of PLS regression. Interestingly, the effect of improvement of model accuracy differ depending on the category of the compound. In this study, it was suggested that metabolites and vitamins had a greater impact on improving model accuracy through machine learning method selection compared to amino acids. These compounds belonged to a group with relatively low accuracy in PLS regression, and it is possible that the effect of improving accuracy is higher for compounds with low accuracy in PLS regression. To demonstrate the improvement in model accuracy, it was demonstrated that methods other than PLS regression, such as XGBoost, can improve modeling accuracy in certain subjects, as shown by the BiP levels in Fig. 4. PLS regression is a linear regression method that selects principal components to capture a linear relationship between predictor and response variables. It reduces multicollinearity and enables accurate models for multivariate data. XGBoost, on the other hand, is a non-linear algorithm that combines decision trees to capture complex patterns. It evaluates feature importance and employs ensemble learning for more accurate predictions. For some categories of compound, XGBoost may outperformed PLS regression due to its ability to capture non-linear relationships, select more appropriate features, and reduce bias and variance through ensemble learning. Hyperparameter tuning was not performed for XGBoost and neural networks in this study, but performing hyperparameter tuning in advanced computational environments may lead to the construction of models that outperform PLS regression, linear regression, and ridge regression.

Through the comprehensive analysis of Raman models of various compounds, it was found that the model accuracy for amino acids was generally high, while the accuracy for vitamins was lower. This discrepancy can be explained by two possible factors. First, the Raman spectra may not detect changes when the compound concentration is too low. Second, the accuracy of the offline measurement, which detecting the compound concentration using LC–MS, may have been compromised at low concentrations, leading to lower accuracy in the Raman measurement model. To improve the accuracy of the Raman measurement model, there is a need to enhance the accuracy of offline measurements. To further improve model accuracy, it is worth considering incorporating information other than Raman spectra into the model. Previous studies have proposed models that combine the computational fluid dynamics models^38^ or include process-related impurities and kinetics of each cultivation data^39^, suggesting that combining this information with Raman spectral data may lead to even higher accuracy models. Additionally, improving the model construction methods is expected to further enhance model accuracy. Narayanan et al. proposed a model construction method that combines Kalman filter^40^, while Poth et al. comprehensively validate algorithms other than those used in this study^41^. It is believed that by extending the model construction methods as reported in these studies, the accuracy of models for compounds with lower accuracy might be further improved. Furthermore, using various variable selection methods in addition to Bayesian optimization, as discussed in the Introduction, may also have the potential to improve accuracy. In this study, the specificity of the measurement was confirmed for glucose by observing the concentration increase upon the addition of a glucose solution. Ideally, an addition experiment should be performed for each compound to confirm specificity. However, by constructing a model for data from multiple cultures with different profiles, specificity can be exhibited. In this study, data from six culture vessels with distinct profiles were used, and the measurement models of each culture vessel were constructed, suggesting the specificity of the measurement results.

Raman spectra primarily detect covalent bonds of compounds in solution, theoretically preventing the detection of metal ions, among others. Interestingly, through exhaustive model construction, models were built for compounds that theoretically could not be detected in Raman spectra, such as hydrogen ions, oxygen, carbon dioxide, and metal ions. Additionally, models were constructed for variables without a physical presence, such as cellular viability. Some compound levels correlated with the values to be measured, indirectly allowing the construction of measurement models (Fig. 4E). For instance, cellular viability is known to correlate with LDH^42^, suggesting the possibility of measuring cellular viability indirectly using LDH level determined with a Raman spectrometer as a proxy. This enables the measurement of not only the concentration of a specific compound but also all variables that characterize a cell culture through certain calculations. It is also possible to estimate the levels of compounds based on the spectral domain and model coefficients used for model construction, contributing to the identification of metabolites that correlate with specific parameters.

This study demonstrates the comprehensive construction of highly precise Raman models for measuring the concentrations of various compounds. This allows continuous acquisition of various culture data using a Raman spectrometer, enabling real-time monitoring and feedback control of culture conditions. While previous Raman measurements and feedback controls focused on glucose and amino acid concentrations, the exhaustive model construction approach may facilitate faster medium development by continuously optimizing a wider range of components.

This technique can be easily expanded to model factors such as omics data. By applying the method used in this study, modeling can be performed for various parameters beyond medium components and metabolites. We successfully constructed a predictive model for BiP, an endoplasmic reticulum stress-related factor, with good precision. Additionally, we constructed a model for oxidative glutathione, an oxidative stress-related factor, suggesting the potential for monitoring not only compound concentrations but also various stress markers. Raman modeling can be considered a feature extraction technique for quantifying culture characteristics, and it is highly compatible with AI-related technologies, which have seen remarkable advancements in recent years. Previous studies predicted transcriptome data from Raman spectra^43^, providing a foundation for predicting multivariate or numerical values. With these technologies, we can develop more comprehensive and accurate models for a broad range of parameters.

## Methods

### Cell substrates and culture methods used

Fed-batch cultures were performed on three clones expressing IgG from serum-free, floating cells derived from CHO-K1 (CCL-61; ATCC, Manassas, VA, USA)^44,45^ in a custom medium (chemically defined) using a 250 mL miniaturized bioreactor (Ambr250; Sartorius, Göttingen, Germany). Cultures were grown at 37 °C, 400 rpm, and maintained below 50% air saturation of dissolved oxygen with a pH of 7.2 (controlled by CO_2_ sparging) for 14 days. Two replicates were cultured for each clone. Cell concentration, viability, metabolites, and antibody levels were monitored over time during culture. Cell density and viability were determined using Vi-CELL (Beckman Coulter). Metabolites were analyzed using Bio Profile FLEX2 (Nova Biomedical, Waltham, MA, USA). Antibody levels were analyzed by high-performance liquid chromatography (HPLC) with a Protein A affinity column (Agilent Technologies, Santa Clara, CA, USA) using a PA ID sensor cartridge Φ2.1 mm × 30 mm (ThermoFisher Scientific, Waltham, MA, USA). Antibody levels were described as titers. Decellularized culture supernatants were stored at − 20 °C and subjected to medium composition analyses by LC–MS and protein-concentration determination by ELISA.

### Raman spectral data acquisition method

During cultivation in Ambr250, 160 µL of the culture broth was sampled and Raman spectra were acquired using a Raman Rxn2 analyzer (Endress Hauser, Reinach, Switzerland). A laser at 785 nm was applied in the flow cell to acquire spectral data ranging in wavenumber from 150 to 3425 cm^−1^. The measurements were performed 10 times for 20 s.

### LC–MS

To perform deproteinization, 60 µL of acetonitrile was added to 40 µL of the culture supernatant. The mixture was vortexed and centrifuged at 10,000 rpm for 15 min. The supernatant (50 µL) after centrifugation was diluted with 450 µL of ultrapure water, and 1 µL was subjected to LC–MS analysis. The Nexera System (Shimadzu, Osaka, Japan) was used for HPLC, and LCMS-8040 (Shimadzu) was used as the mass spectrometer. Acetonitrile was used as the mobile phase. The analytical column used was Discovery HS F5 (2.1 mm × 150 mm, 3 µm) (Sigma-Aldrich, St. Louis, MO, USA), and the mobile phases used were 0.1% formic acid–water and 0.1% acetonitrile. Compound identification and quantitation were performed using the LC/MS/MS method package cell-culture profiling (Shimadzu) with data reported as relative concentrations. The acquired data were standardized and used as objective variables for model construction.

### ELISA

BiP levels were measured by ELISA using the GRP78/BiP ELISA Kit (#AD1-900-214; Enzo Life Sciences, Inc., Farmingdale, NY, USA). Primary antibodies (50 µL) were added to 100 µL of the culture supernatant and gently shaken for 60 min at room temperature. Subsequently, 50 µL of secondary antibody was added to the post-reaction solutions and gently shaken for 60 min at room temperature. The reactants were discarded, washed at least three times with wash buffer, and 200 µL of TMB solution was added and shaken for 30 min to develop color. Finally, 50 µL of stop solution was added to stop the reaction, and the absorbance at 450 nm was measured. BiP levels were quantified from the calibration curves measured using standard solutions.

### Model building

All the calculations were performed on a Linux server having a dual Intel® Xeon® E5-2667 v4 processor (3.20 GHz), 125 GB RAM, with Ubuntu 18.04.1 LTS operating system. Python 3.8.8 was used to build the model. Data frames were generated with Raman spectral data as explanatory variables and culture profile data as objective variables. For the Raman spectral data, the minimum wavenumber was set as 100 cm^−1^, and the maximum was set as 3425 cm^−1^. The spectral range was defined with a minimum value of 125 cm^−1^ and a maximum value of 3325 cm^−1^. Among the 78 sets of data, data acquired from five reactors were used as training data, and data acquired from one of the remaining reactors were used as test data. The data to be measured were standardized, and for Raman spectrum data, a dataset was created by preprocessing the data in the following order: untreated, standardized, smoothed (moving average of 10 points), first-order differential, and second-order differential. Machine learning algorithms such as PLS regression, linear regression, ridge regression, XGBoost, and neural network were used with methods from the scikit-learn library (version 0.24.1) for PLSRegression, LinearRegression, Ridge, and MLPRegressor, and methods from the XGBoost library (version 1.7.1) for XGBRegressor. For the hyperparameters of MLPRegressor and XGBRegressor, the defaults of scikit-learn were used. Bayesian optimization was performed using the GPyOpt package (version 1.2.6) with up to 20 attempts. Model performance was assessed using R^2^ and RMSE as indicators^46^. R^2^ measures the proportion of the variance in the dependent variable that can be explained by the independent variables. RMSE calculates the average deviation between predicted and actual values, providing an overall measure of accuracy. Each formula is shown below, where ${y}_{i}$ represents the actual value, ${\widehat{y}}_{i}$ represents the predicted value and ${\overline{y} }_{i}$ represents the average of actual values:

$${R}^{2}= 1-\frac{{\sum }_{i=1}^{m}{({y}_{i}-{\widehat{y}}_{i})}^{2}}{{\sum }_{i=1}^{m}{({y}_{i}-{\overline{y} }_{i})}^{2}}$$

$$RMSE= \sqrt{\frac{1}{m}{\sum }_{i=1}^{m}{({y}_{i}-{\widehat{y}}_{i})}^{2}}$$

### Supplementary Information

Supplementary Figures.

## Data Availability

The data that support the findings of this study are available from the corresponding author upon reasonable request. Additional data are available in the supplementary material of this article.
