# Supplementary material for: Comprehensive modeling of cell culture profile using Raman spectroscopy and machine learning
Source: Sci Rep. 2023 Dec 9;13:21805. doi: 10.1038/s41598-023-49257-0 (PMC10710501; doi:10.1038/s41598-023-49257-0)
Supplement: Supplementary file 1 — Supplementary Figures. [file 41598_2023_49257_MOESM1_ESM.pdf]

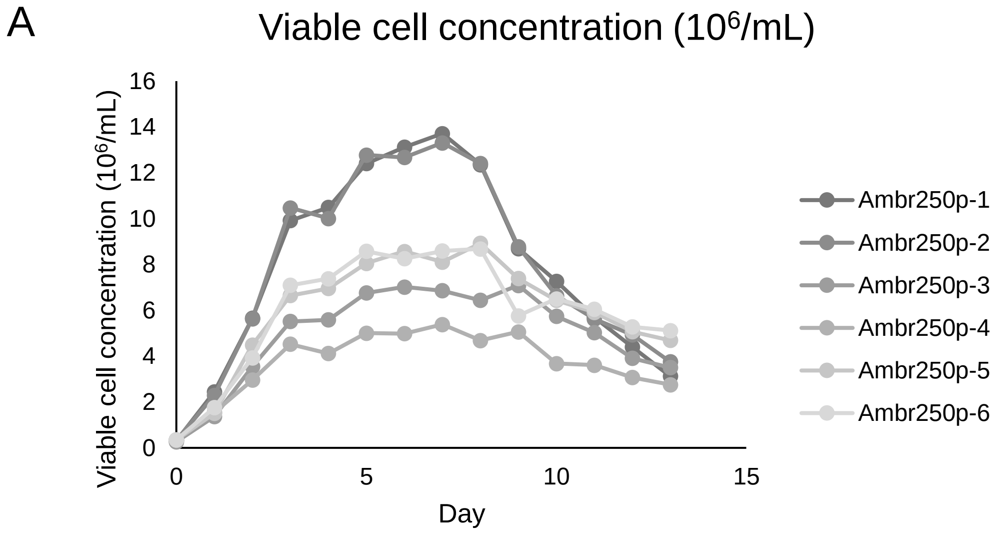

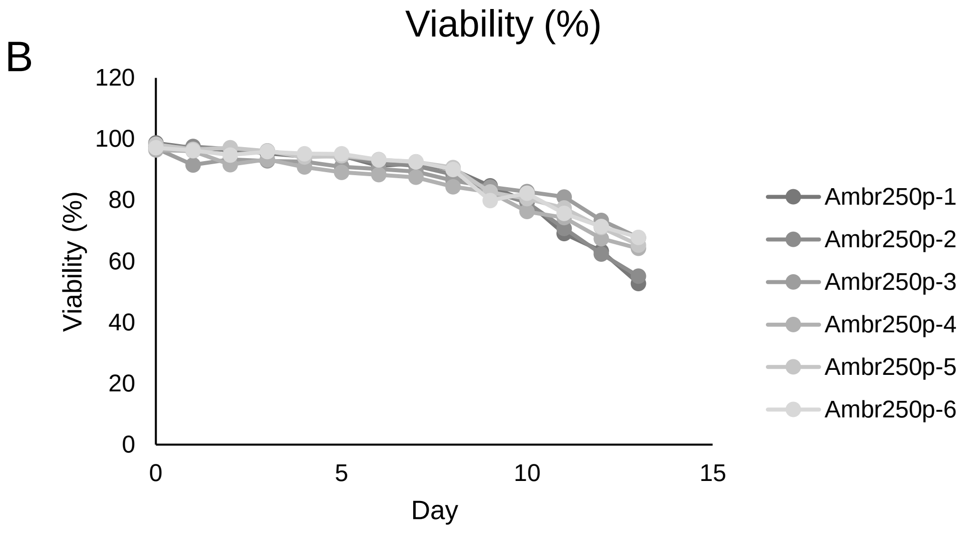

Supplemental Figure 1: Cell culture results (cell growth and viability)
Cell culture results of 6 reactors (Ambr250p-1~6) (A) Viable cell concentration (B) Cell viability

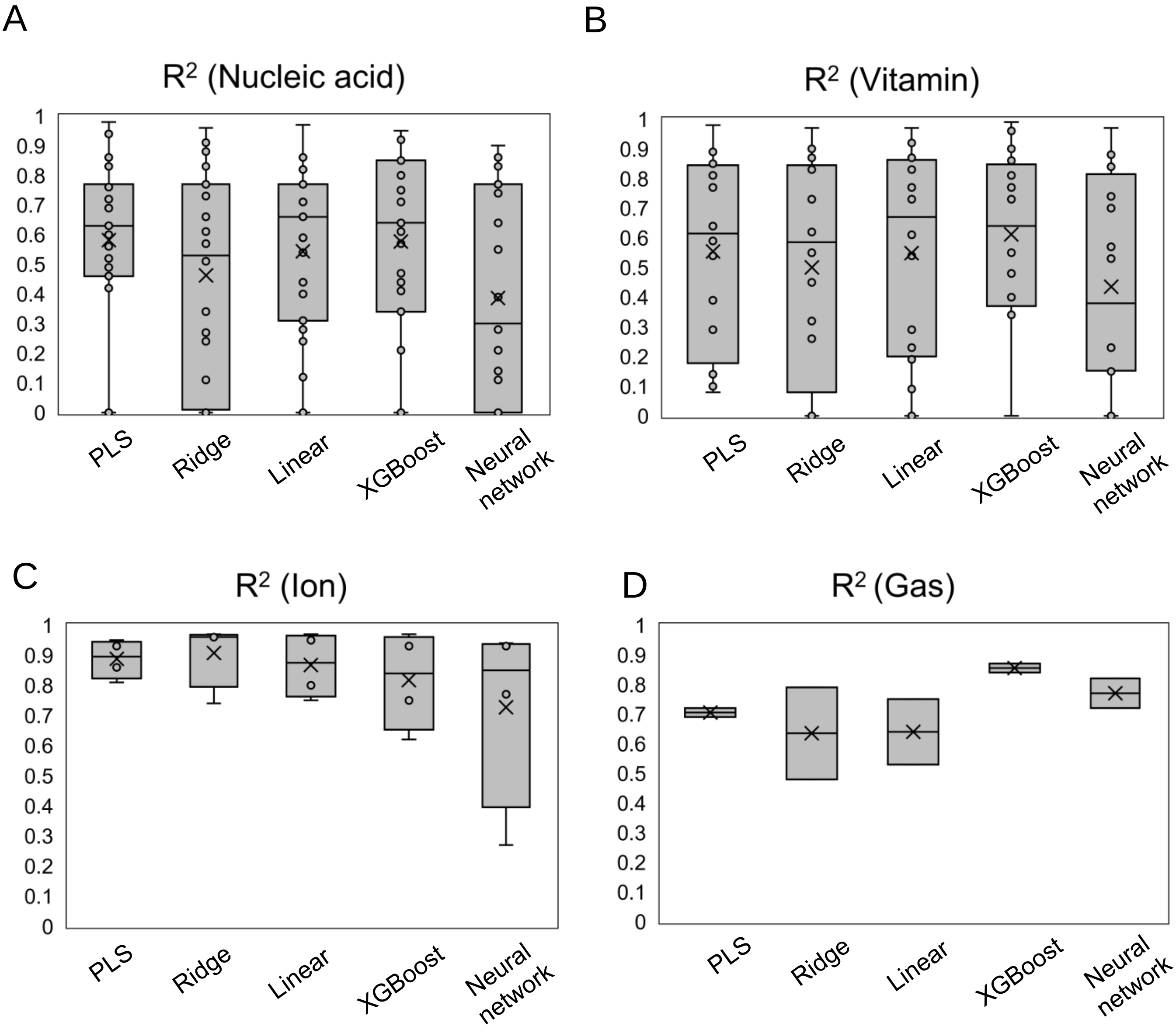

Supplemental Figure 2: Comparing the performance of Raman measurement models with different machine-learning techniques.

Raman measurement models were constructed for each parameter using various machine-learning techniques, and their performance was evaluated. The R^2 values were plotted as performance assessment indices, and the means and error ranges were shown using box-and-whisker plots. In this plot, an “x” (cross) represents the mean, the box represents the interquartile range, a line inside the box represents the second quartile, and the whiskers represent the minimum (1.5 times the interquartile range below the first quartile) and maximum (1.5 times the interquartile range above the third quartile) values of the data. The graph is divided into compound categories: (A) Nucleic acids, (B) Vitamins, (C) Ions including Na^+, K^+, Ca^{2+}, and (D) Dissolved O_2 and CO_2.
